# *APOE2* mitigates disease-related phenotypes in an isogenic hiPSC-based model of Alzheimer’s disease

**DOI:** 10.1038/s41380-021-01076-3

**Published:** 2021-04-09

**Authors:** Nicholas Brookhouser, Sreedevi Raman, Carlye Frisch, Gayathri Srinivasan, David A. Brafman

**Affiliations:** 1 School of Biological and Health Systems Engineering, Arizona State University, Tempe, AZ USA; 2 Graduate Program in Clinical Translational Sciences, University of Arizona College of Medicine-Phoenix, Phoenix, AZ USA

**Keywords:** Stem cells, Cell biology

## Abstract

Genome-wide association studies (GWAS) have identified polymorphism in the Apolipoprotein E gene (*APOE*) to be the most prominent risk factor for Alzheimer’s disease (AD). Compared to individuals homozygous for the *APOE3* variant, individuals with the *APOE4* variant have a significantly elevated risk of AD. On the other hand, longitudinal studies have shown that the presence of the *APOE2* variant reduces the lifetime risk of developing AD by 40 percent. While there has been significant research that has identified the risk-inducing effects of *APOE4*, the underlying mechanisms by which *APOE2* influences AD onset and progression have not been extensively explored. In this study, we utilize an isogenic human induced pluripotent stem cell (hiPSC)-based system to demonstrate that conversion of *APOE3* to *APOE2* greatly reduced the production of amyloid-beta (Aβ) peptides in hiPSC-derived neural cultures. Mechanistically, analysis of pure populations of neurons and astrocytes derived from these neural cultures revealed that mitigating effects of *APOE2* are mediated by cell autonomous and non-autonomous effects. In particular, we demonstrated the reduction in Aβ is potentially driven by a mechanism related to non-amyloidogenic processing of amyloid precursor protein (APP), suggesting a gain of the protective function of the *APOE2* variant. Together, this study provides insights into the risk-modifying effects associated with the *APOE2* allele and establishes a platform to probe the mechanisms by which *APOE2* enhances neuroprotection against AD.

## Introduction

Although a vast majority of AD cases are sporadic, numerous genetic risk factors have been identified that contribute to lifetime risk of developing the disease [1]. While not deterministic, the polymorphisms in the *APOE* gene have been established to be the most potent modulator of late onset, sporadic, AD. In the central nervous system, APOE is generated and secreted principally by astrocytes and microglia, and, to a lesser degree, neurons. In the brain, ApoE functions to transport cholesterol and other lipids to neurons and plays important roles as it relates to neuronal growth, synaptic plasticity, and membrane repair [2, 3]. Human APOE has three major isoforms, APOE2, APOE3, and APOE4, which differ by two amino acid substitutions at residues 112 and 158 in exon 4—APOE2 (Cys112, Cys158), APOE3 (Cys112, Arg158), APOE4 (Arg112, Arg158). With respect to AD, compared with individuals homozygous for the *APOE3* allele (referred to as the “risk neutral” allele), heterozygosity for the ε4 allele increases AD risk by 3 fold, and homozygosity for the ε4 allele increases risk over 10 fold [4, 5]. Conversely, individuals with the ε2 allele (referred to as the “protective” allele) are 40% less likely to develop AD [4, 5]. In fact, recent epidemiological studies have revealed that *APOE2* homozygotes have an exceptionally low likelihood of AD [6]. As such, understanding the mechanisms that underlie the association between *APOE2* and protection against neurodegeneration may provide new therapeutic targets.

Extensive work has examined the role of *APOE4* in contributing to AD onset and age-related progression. Previous studies have established that the *APOE4* variant increases likelihood of developing AD in a dose dependent manner as the number of *APOE4* alleles increases, while greatly reducing the mean age of onset [7]. In addition, it has been demonstrated that *APOE4* modulates the formation of amyloid plaques and neurofibrillary tangles [5], two pathological hallmarks of AD. Moreover, numerous studies have identified several amyloid-dependent and -independent mechanisms to explain the risk-inducing effects of *APOE4* [8, 9]. By comparison, there is significantly less research that has examined the protective effects of *APOE2*. In general, the presence of an *APOE2* allele has been associated with decreased AD-related neuropathology, age-associated cognitive decline, and greater cortical thickness [10, 11]. Along similar lines, several studies have suggested that *APOE2* modulates Aβ deposition, clearance and degradation, antioxidant and anti-inflammatory activity, and neuronal glucose metabolism [3, 12, 13]. Finally, while the risk modifying effects of *APOE* have been largely studied in the context of late-onset AD (LOAD) patient populations, there are some studies that have examined the effect of *APOE* genotype in the context of familial AD (fAD)-related mutations. Consistent with what is observed in LOAD subjects, the presence of *APOE4* significantly reduces the age of disease onset whereas carriers of *APOE2* have delayed age of onset [14–18].

Animal models have provided important insights to explain the risk-modulating effects of various *APOE* isoforms [8, 9, 19]. However, the inherent complexities of such model systems made it difficult to make definite mechanistic links between *APOE* genotype and AD-related phenotypes. In addition, the multi-cellular composition of the in vivo environment does not allow for the identification of cell-autonomous versus cell-non autonomous aspects of such relationships. Due to the limitations of current animal-based models, complimentary human cell-based models are needed to study the biochemical, molecular, and cellular mechanisms that underlie risk-modifying effects of various *APOE* isoforms. To date, most studies have been limited to immortalized cell lines, which are karyotypically abnormal with non-physiological dosage at disease-relevant genes [20]. Conversely, studies of normal human cells from cadaveric tissue samples with respect to *APOE2* are often limited to small sample sizes (given the low allele frequency), making it challenging to assign observed phenotypes to a specific *APOE* genotype or as a by-product of the genomic diversity present across individuals.

Over the past several years, hiPSC-based models have been used extensively by numerous groups to study AD [21, 22] and, to a lesser extent, the influence of *APOE* on disease-relevant phenotypes [23, 24] in a simplified and accessible system. However, the analysis of the phenotypic effects of specific risk factors, such as those in the *APOE* locus, has been confounded by the genetic and epigenetic differences inherent in the individual hiPSC lines derived from distinct patient genomes. As such, the use of isogenic cell lines with identical genetic backgrounds that only differ with respect to individual variants has become essential making definitive genotype-to-phenotype relationships as it relates to the modeling of AD-related mutations and risk factors. To this end, we recently reported the development of a series of methods that employ a transient reporter for editing enrichment (TREE), which allows for the generation of isogenic hiPSC lines with clonal homozygous editing efficiencies approaching 90% [25, 26]. In this study, we leverage this technology to characterize AD-related phenotypes in neural cultures derived from hiPSC lines harboring early onset, familial AD (fAD), mutations with an *APOE3* genotype and gene edited isogenic *APOE2* matched pairs. Detailed phenotypic analysis of these cultures revealed that conversion of *APOE3* to *APOE2* significantly reduced Aβ secretion. In addition, we found that *APOE2* significantly decreased levels of phosphorylated tau in isogenic cultures that showed a concomitant decrease in the Aβ42/40 ratio. Furthermore, we developed a cell separation protocol to study AD phenotypes in pure populations of neurons and astrocytes. This analysis revealed that the protective effect conferred by *APOE2* had cell autonomous and non-autonomous contributions as cultures consisting exclusively of neurons displayed a reduction, but not complete abrogation of the *APOE2* protective effect. Mechanistically, we demonstrate that *APOE2* contributes, in part, to the mitigation of these AD-related phenotypes through changes in amyloid precursor protein (APP) processing. Overall, a more thorough understanding of the mechanisms by which *APOE2* enhances neuroprotection against AD will have a significant translational impact on the design of therapeutic interventions.

## Materials and methods

### Human iPSC culture

hiPSCs were maintained in mTeSR1 medium (Stemcell Technologies) on feeder-free Matrigel (Corning)-coated plates. Subculture was performed every 3 days using Accutase (Life Technologies) in mTeSR1 medium supplemented with 5 µM Rho kinase inhibitor (ROCKi; Y-27632 [Tocris Bioscience]). Control and AD-patient hPSCs were generated from dermal fibroblasts as previously described [27]. hiPSC lines described in the manuscript are as follows:

**Table:** 

Cell line	Disease status	MMSE	Mutation	Citation
hPSC Line 1 *APOE3/3*	Familial AD	n/a	APP V717I	Muratore et al. 2014
hPSC Line 1 Isogenic *APOE2/2*	Familial AD	n/a	APP V717I	Brookhouser et al. 2020
hPSC Line 2 *APOE3/3*	Familial AD	n/a	APPdp	Israel et al. 2012
hPSC Line 2 Isogenic *APOE2/2*	Familial AD	n/a	APPdp	This paper
hPSC Line 3 *APOE3/3*	Familial AD	n/a	PSEN1 A246E	https://biomanufacturing.cedars-sinai.org/product/cs40ifad-nxx/
hPSC Line 3 Isogenic *APOE2/2*	Familial AD	n/a	PSEN1 A246E	Brookhouser et al. 2020
hPSC Line 4 *APOE3/3*	Non-demented control	30	n/a	Hjelm et al. 2011
hPSC Line 4 Isogenic *APOE2/2*	Non-demented control	n/a	n/a	Brookhouser et al. 2020

### Neuronal differentiation of hiPSCs

hiPSCs were differentiated to hNPCs as previously described with some modifications [28]. Briefly, to initiate neural differentiation hiPSCs were cultured in feeder-free conditions (Matrigel^TM^ [BD Biosciences]; mTeSR1™ Medium [Stemcell Technologies]) for a minimum of two passages. Cells were then detached with Accutase (ThermoFisher) and resuspended in mTeSR1 media supplemented with 5 µM Y-27632. Next, 1-2 × 10^6^ cells were pipetted to each well of a 6-well ultra-low attachment plate (Corning). The plates were then placed on an orbital shaker set at 95 rpm in a 37 °C/5% CO_2_ tissue culture incubator. The next day, the cells formed spherical cultures (embryoid bodies [EBs]) and the media was changed to neural induction media (NIM) [1X DMEM-F12 (ThermoFisher), 0.5% (v/v) N2 supplement (ThermoFisher), 1% (v/v) B27 supplement (ThermoFisher), 1% (v/v) GlutaMAX supplement (ThermoFisher), 1% (v/v) Penicillin Streptomycin, 50 ng/ml recombinant human Noggin (R&D Systems), 0.5 µM Dorsomorphin (Tocris Bioscience)]. Half of the media was subsequently changed every day. After 6 days in suspension culture, the EBs were then transferred to a 10 cm dish (1-2 6 wells per 10 cm dish) coated with Matrigel™. The plated EBs were cultured in NIM for an additional 5–7 days. Cells were then plated on surfaces that had to be coated first with poly-L-ornithine (PLO; Sigma) and then with mouse laminin (LN; 4 µg/mL; ThermoFisher). For routine maintenance, hNPCs were passaged onto LN-coated plates at a density of 1-5 × 10^4^ cells/cm^2^ in neural expansion media (NEM; [1X DMEM-F12, 0.5% (v/v) N2 supplement, 1% (v/v) B27supplement, 1% (v/v) GlutaMAX supplement, 1% (v/v) Penicillin Streptomycin, 30 ng/ml FGF2, and 30 ng/ml EGF]). hNPCs were expanded and seeded on LN-coated microcarriers (MCs) in ultra-low attachment 6-well plates (Corning) at a density of 3 × 10^6^ cell per well and 1 mg/mL MCs. The plates were placed under static conditions with 2 mL of NEM in each well to allow for cell attachment on MCs for 12 h after which an additional 2 mL of NEM was added to the wells. The plates were placed on an orbital shaker (Dura-Shaker, VWR) at 95 RPM. Three-fourths of the media (~3 mL) was changed after 24 h of culture to remove the ROCKi and half of the media (~2 mL) was changed every day thereafter. HNPCs were expanded on LN-coated MCs for 4–5 days until 80–90% confluent after which the media was switched to neuronal differentiation media (NDM); [1X DMEMF12, 0.5% (v/v) N2 supplement, 1% (v/v) B27 supplement, 1% (v/v) GlutaMAX, 1% (v/v) Penicillin Streptomycin, 20 ng/ml BDNF (Stemcell Technologies), 20 ng/ml GDNF (Stemcell Technologies)]. Cells were differentiated for a minimum of 30 days prior to replating for analysis.

### Coating of microcarriers (MCs) with laminin

To coat MCs (Corning Enhanced Attachment Microcarriers) with LN, MCs were suspended in 4 µg/mL PLO solution and incubated overnight at 37 °C, after which the MCs were washed twice with PBS. The MCs were then coated with 4 µg/mL LN solution at 37 °C overnight. Coated MCs were washed once with PBS and once with culture media prior to use.

### Dissociation of neurons from microcarriers

Cells were incubated in Accutase for 5 min and the aggregates were pipetted up and down gently using a P1000 to break apart large aggregates. Next, cells were detached from the MCs by incubating in a papain solution containing Earle’s balanced salt solution (Alfa Aesar), 30 U/mL papain (Worthington), 1 mM L-Cysteine, 22.5 mM D-glucose, 26 mM NaHCO_3_ and 125 U/mL DNase (Roche) for 70 min at 37 °C and then triturated with an inhibitor solution containing 1 mg/mL ovomucoid inhibitor (Roche) and 1 mg/mL BSA (Sigma) after which the cell-suspension was passed through a 40 μm cell strainer to remove the MCs and obtain a single cell suspension [29].

### Isolation of pure neuronal and astrocytic populations using MACS

Following dissociation from MCs, cells were placed at 37 °C for 1 h to allow recovery of cell surface proteins. Cells were washed with MACS buffer and resuspended at a concentration of 1 × 10^8^ cells/mL in MACS buffer in a 5 mL round bottom tube. Biotinylated human anti-CD44 antibody (BD Biosciences, Cat # 550989) was added according to manufacturer recommendations and incubated on ice for 15 min. Next, streptavidin conjugated magnetic beads were added following manufacturer protocol, and allowed to incubate on ice for 15 min. The tube was then placed in the magnet (BioLegend) and incubated for 5 min at room temperature (RT). Following the incubation, CD44 negative (CD44−) cells were transferred to a new tube while CD44 positive (CD44 + ) cells remained in the tube bound to the magnet. The tube was then removed from the magnet, and cells were resuspended in astrocyte media (ScienCell) and plated in Matrigel coated wells and expanded for downstream assays. CD44− neurons were plated in a Matrigel coated 24-well plate at 1 × 10^6^ cells per well for downstream assays.

### Immunofluorescence

Cultures were gently washed twice with PBS prior to fixation for 15 min at RT with BD Cytofix Fixation Buffer (BD Biosciences). The cultures were then washed twice with PBS and permeabilized with BD Phosflow Perm Buffer III (BD Biosciences) for 30 min at 4 °C. Cultures were then washed twice with PBS. Primary antibodies were incubated overnight at 4 °C and then washed twice with PBS at room temperature. Secondary antibodies were incubated at RT for 1 h. DNA was stained with Hoechst 33342 (2 μg/mL; Life Technologies) for 10 min at RT and then washed twice with PBS. Antibodies were used at the following concentrations: TUJ1 (1:1000; Fitzgerald Industries International, Cat# 10R-T136A), MAP2 (1:500; Millipore, Cat# AB5622), NEUN (1:500; Millipore, Cat# MAB377), GFAP (1:500; Abcam, Cat# AB7260), S100B (1:500; Sigma, Cat# S2532), AFP (1:50; Santa Cruz Biotechnology, Cat# sc-8399), SMA (1:50; Santa Cruz Biotechnology, Cat# sc-53015), Alexa 488 donkey anti-mouse (1:500; ThermoFisher, Cat# A-21202), and Alexa 488 donkey anti-rabbit (1:500; ThermoFisher, Cat# A-21206), Alexa 647 donkey anti-mouse (1:500; ThermoFisher, Cat# A-31571), and Alexa 647 donkey anti-rabbit (1:500; ThermoFisher, Cat# A-31573).

### Tri-lineage differentiation of edited hiPSCs

hiPSCs were harvested using Accutase and plated on ultra-low attachment plates in mTeSR1 medium. The following day, media was changed to differentiation medium (DM; DMEM/F12, 20% FBS (ThermoFisher), 1% Pen/Strep). After 5 days, EBs were plated on Matrigel-coated plates and cultured with DM. After 21 days in DM, cells were fixed, permeabilized, and stained for germ layer markers.

### Flow cytometry analysis of CD44 expression

Cells were dissociated with Accutase for 10 min at 37 °C, triturated, and passed through a 40 μm cell strainer. Cells were then washed twice with flow cytometry buffer (BD Biosciences) and resuspended at a maximum concentration of 1 × 10^6^ cells per 100 μL. Cells were stained with PE-Mouse anti-human CD44 or isotype control (PE-Mouse IgG1,κ, BD Biosciences, Cat# 555749) antibodies for 1 h on ice. Cells were washed twice with flow cytometry buffer prior to analysis. Flow cytometry analysis was performed on an Accuri C6 flow cytometer (BD Biosciences). Flow cytometry files were analyzed using FlowJo (BD).

### Fluorescence microscopy

All imaging was performed on a Nikon Ti2-Eclipse inverted microscope with an LED-based Lumencor SOLA SE Light Engine using a Semrock band pass filter.

### Measurement of Aβ40, Aβ42, sAPPα, and sAPPβ

Neurons were seeded in a 24-well plate at a density of 1 × 10^6^ cells per well. Six-day conditioned media was collected and Aβ levels were measured using the R&D Systems Human Amyloid Beta 40 and 42 ELISA Kits. Six-day conditioned media was used to quantify sAPPα and sAPPβ using highly sensitive ELISA kits (IBL). Cell lysate was collected for normalization to cellular protein amounts.

### Measurement of total APP and APP-βCTF in cell lysates

Neurons were seeded in a 24-well plate at a density of 1-2 × 10^6^ cells per well, cultured for 6 days and subsequently lysed in RIPA buffer containing protease inhibitors (ThermoFisher). Total APP and APP-βCTF levels in the cell lysates were measured using commercially available ELISA kits from ThermoFisher and IBL respectively.

### Measurement of pTau and Total Tau in cell lysates

Cell lysates were analyzed using Human Tau ELISA kits (ThermoFisher) to determine pTau and total tau levels.

### Measurement of APOE

Six-day conditioned media was collected from neurons and astrocytes seeded in a 24-well plate. APOE levels were measured using a human APOE ELISA kit (ThermoFisher) and were normalized to total cellular protein level.

### Calcium transient imaging

Calcium imaging was performed on cells seeded on a 30 mm glass bottom dish (TED PELLA) coated with Matrigel. Neurons were incubated with 1 µM Fluo-4-AM (Invitrogen) and 0.02% Pluronic™ F-127 in DMEM for 30 min at 37 °C. To reduce dye associated cytotoxicity, astrocytes were incubated with 0.5 µM Fluo-4-AM (Invitrogen) and 0.02% Pluronic™ F–127 in DMEM for 15 min at 37 °C. Cells were then washed once with HEPES-buffered Tyrode’s solution (Alfa Aesar), and allowed to de-esterify at RT in the dark for 20 min prior to imaging on a Nikon Ti2-Eclipse inverted microscope. Fluorescent time-lapse images for both neurons and astrocytes were acquired (20x objective) using an 80 ms exposure time at one frame per second for 300 s. Calcium spike traces were generated by quantifying the mean pixel intensity of manually identified regions of interest using Fiji [30].

### β-amyloid uptake assay

FAM-labelled β-amyloid peptide (Aβ-FAM 1-42; Anaspec) was reconstituted as per manufacturer’s instructions. Briefly, a minimal volume of 1%NH_4_OH was added to the peptide and immediately diluted to a 1 mg/mL solution in PBS prior to storage as single-use aliquots at −80 °C. To measure Aβ uptake, astrocytes and neurons seeded in a 24-well plate were treated with 500 nM Aβ-FAM for 24 h. For flow cytometry analysis, cells were washed with cold PBS and dissociated using 0.25% Trypsin-EDTA (ThermoFisher) for 5 min at 37 °C to remove any surface-bound peptide. Samples were filtered using a 40 µm cell strainer and placed on ice till analysis. An Accuri C6 flow cytometer (BD Biosciences) was to quantify the median fluorescence intensity (MFI). Following background correction using untreated cell MFI, signal was normalized to bulk endocytosis MFI from cells treated with 50 ug/mL Dextran, Alexa Fluor™ 647 (10,000 MW; Invitrogen) for 1 h at RT.

### Surface receptor expression measurement

Astrocytes seeded on a 6-well plate were dissociated non-enzymatically by gentle scraping following incubation with Versene (Gibco) for 15 min at 37 °C. Subsequently, cells were washed twice in stain buffer (BD Biosciences). Cells were then incubated with primary antibodies (and appropriate isotype controls) specific to the cell surface epitope of the LRP-1 (Biotechne) or LDLR (BD Biosciences) for 30 min on ice. Following two washes in stain buffer, 5000–10,000 live cells were acquired on an Accuri C6 flow cytometer (BD Biosciences).

### RNA-seq analysis

Single end sequencing was performed at BGI Americas Corporation using BGISEQ-500 for a 50 bp run as described previously [31]. Reads were subsequently mapped to the hg19 human reference genome using HISAT2 [32]. Differential analysis was performed using DEseq2 algorithms [33]. Gene ontology analysis was performed using lists of differentially expressed genes using PANTHER v16 [34].

### Statistical analysis

Differences between groups was determined using unpaired two-sided *t* test (Welch’s *t* test for comparison of unequal sample sizes) with *P* < 0.05 considered to be significant using GraphPad Prism version 9.0.0 for macOS. Unless otherwise noted, all data are displayed as mean ± s.e.m.

## Results

### Generation of an isogenic hiPSC-derived cell culture model of AD to study effects of *APOE2*

**Fig. 1:**
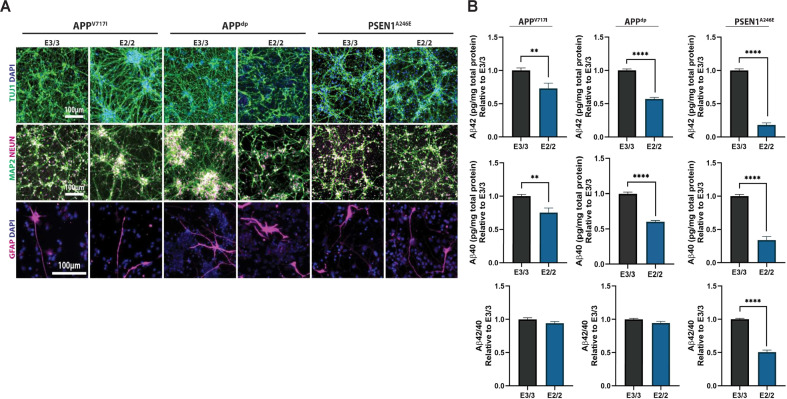
Isogenic hiPSC-derived model of AD reveals isoform-specific effects of APOE2. **A** Characterization of hiPSC-derived neural cultures by immunofluorescence for neuronal marker TUJ1, mature neuron markers MAP2 and NEUN, and astrocyte marker GFAP. **B** Quantification of secreted soluble Aβ levels in cell supernatant normalized to cellular protein amounts, relative to corresponding E3/3 cultures. *n* = 15~16 from 3 independent differentiations; *p < 0.05, **p < 0.01, ***p < 0.001, ****p < 0.0001.

We have previously described a highly efficient method for the generation of isogenic hiPSCs [25]. To that end, we applied these strategies in hiPSC lines homozygous for the *APOE3* allele to generate isogenic pairs with an *APOE2* genotype. For this study, we focused on the analysis of three isogenic pairs with mutations in APP (APP^V717I^, APP^dp^) and PSEN1 (PSEN1^A246E^). hiPSC lines harboring these mutations have been previously published and shown to produce robust phenotypes in vitro when differentiated to neural cultures [21, 22, 24], making them advantageous for detecting phenotypic differences that may arise as a result of genome modification. In addition, we analyzed one isogenic pair derived from a non-demented control (NDC) individual identified by clinical studies and post-mortem histopathological examination negative for major neurological and neuropathological conditions [35]. Characterization of isogenic APP^V717I^, PSEN1^A246E^, and NDC lines has been described previously [25], and full characterization of the APP^dp^ *APOE2* line was performed prior to phenotypic analysis in this study (Supplementary Fig. S1). Specifically, all clonal isogenic hiPSC lines displayed a characteristic hiPSC morphology, high expression of pluripotency markers OCT4, NANOG, and SOX2, ability to differentiate in vitro into cell types representative of three primitive germ layers, and a normal euploid karyotype. We then used scalable, microcarrier (MC)-based differentiation strategies developed in our laboratory [31] to generate neural cultures from each isogenic pair. Briefly, multipotent human neural cells (hNPCs) were established using our previously described methods [36]. HNPCs were then seeded on laminin (LN)-coated microcarriers in 6-well ultra-low attachment plates and the medium was changed to a neural differentiation medium. After a minimum of 30 days of differentiation, cultures were dissociated into single cells and replated onto Matrigel-coated tissue culture plates. Analysis of these cultures revealed a large percentage of cells that expressed the mature neuronal markers TUJ1, MAP2, and NEUN (Fig. 1A). In addition, these differentiation conditions also resulted in the generation of numerous supporting GFAP + astrocytes (Fig. 1A), which is important because many AD-related neuronal phenotypes modulated by *APOE* are mediated by their interactions and signals from astrocytes [37, 38]. Overall, neural differentiations as measured by TUJ1, MAP2, and NEUN expression were consistent across lines, isogenic pairs, and independent differentiations, suggesting no change in differentiation potential as a result of genome modification (Supplementary Fig. S2).

### *APOE2* modulates AD-related phenotypes in hiPSC-derived neural cultures

In the strictest form of the amyloid cascade hypothesis, generation and subsequent oligomerization of Aβ is the primary step that leads to elevated phosphorylated tau (p-tau) and subsequent synaptic and neuronal loss. To that end, several studies have suggested that the AD-risk modulating effects of *APOE* occur at multiple levels in the amyloid cascade [8, 9]. To investigate the extent to which Aβ secretion is influenced by *APOE2*, we measured the neural cultures for levels of Aβ40, and Aβ42 released into the extracellular medium by ELISA. This analysis revealed that conversion of *APOE3* to *APOE2* significantly reduced levels of secreted aggregation-prone Aβ42 in cell supernatant (Fig. 1B). In addition, *APOE2* neurons secreted less Aβ40 peptide compared with *APOE3* matched pairs (Fig. 1B), suggesting *APOE2* plays a critical role in the regulation of Aβ peptide generation and, consequently, in lowering total amyloid burden. Finally, we assessed the Aβ42/40 ratio in the neural cultures and found the reduction in amyloid significantly reduced the ratio in *APOE2* neurons harboring the PSEN1^A246E^ mutation; however, had no effect on the ratio in neural cultures derived from APP mutants (Fig. 1B). As it has been reported that the APP mutations can specifically increases Aβ42 generation, we speculate that the conversion to *APOE2* may not be sufficient to alter the ratio in these mutants due to production of such high levels of Aβ42 [24]. Finally, the introduction of *APOE2* into non-demented control (NDC) hiPSCs did not reduce Aβ levels or the Aβ42/40 ratio in the neural cultures (Supplementary Fig. S3a). Taken together, these data suggest that conversion of *APOE3* to *APOE2* significantly moderated levels of Aβ that were elevated by fAD-related mutations (Supplementary Fig. S4).

**Fig. 2:**
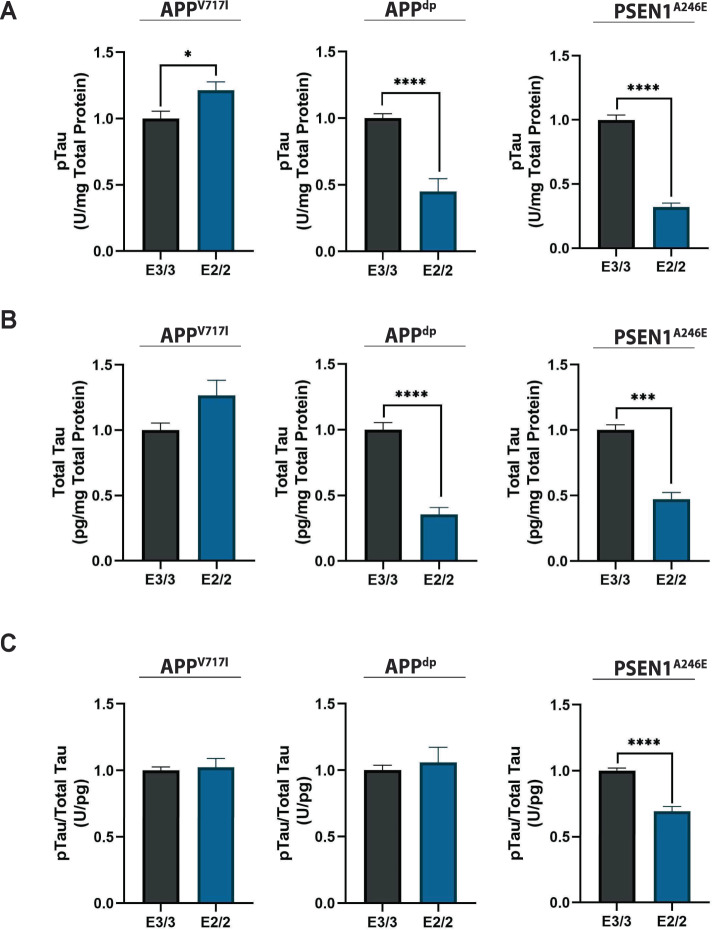
Isoform-specific effect of APOE2 on tau protein. **A** Quantification of phosphorylated-tau in cell lysates normalized to total protein. **B** Quantification of total tau normalized to total protein in lysate. **C** Corresponding ratio of phosphorylated-tau protein to total tau. *n* = 14~15 from 3 independent differentiations; *p < 0.05, **p < 0.01, ***p < 0.001, ****p < 0.0001.

Next, we investigated the effect of *APOE2* on levels of p-tau in isogenic fAD and NDC neural cultures. As such, we measured tau phosphorylation at Thr231, a tau phosphoepitope, (Fig. 2A**;** Supplementary Fig. S3b, left panel) as well as total tau in neural cell lysates (Fig. 2B**;** Supplementary Fig. S3b, center panel). Interestingly, we found that relative levels of phosphorylated tau to total tau were significantly reduced only in *APOE2* isogenic cultures that showed a decrease in Aβ42/40 ratio (i.e., PSEN1^A246E^; Fig. 2C**;** Supplementary Fig. S3b, right panel). Overall, our results are consistent with emerging evidence that tau pathology is largely driven by the Aβ42/40 ratio and not total amyloid levels [39].

### Astrocytes mediate the mitigating effects of APOE2 on amyloid levels in hiPSC-derived neurons

**Fig. 3:**
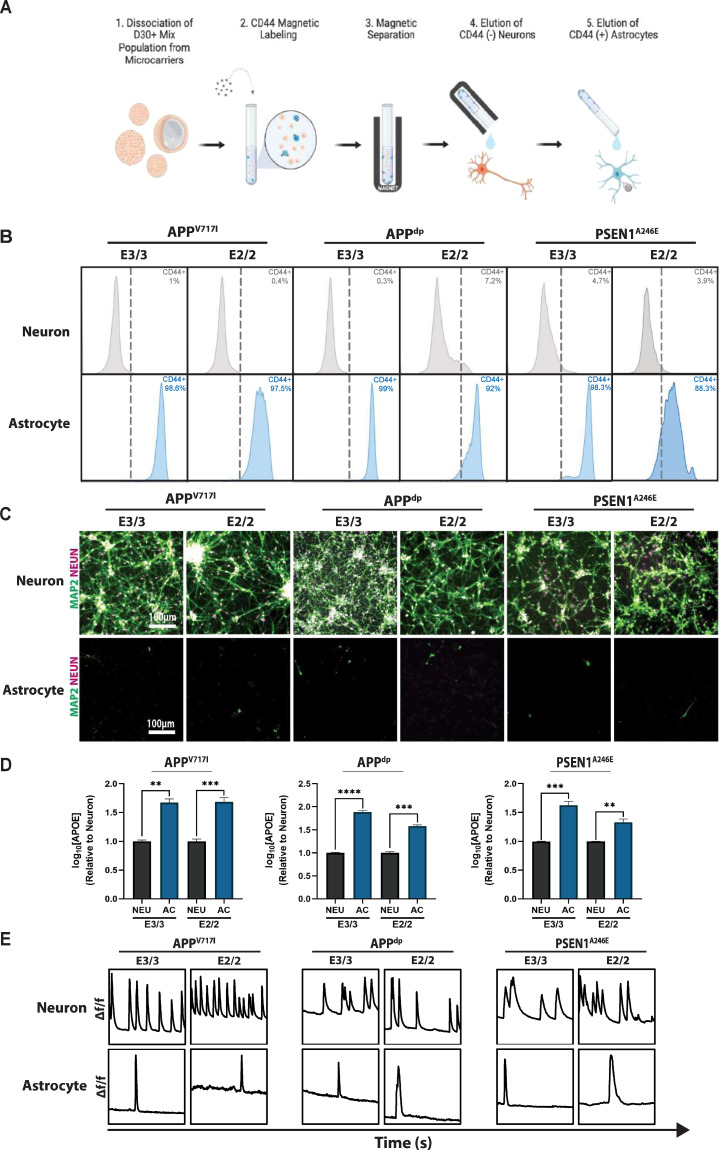
Isolation of pure populations of neurons and astrocytes from mixed cultures. **A** Schematic of magnetic-activated cell sorting (MACS) used to isolate pure neuronal and astrocytic cell populations. Schematic was generated using BioRender. **B** Representative flow cytometry analysis of CD44 expression in separated CD44− neurons and CD44 + astrocytes. **C** Immunofluorescence showing expression of mature neuron markers MAP2 and NEUN in neuron, but not astrocyte populations. **D** Quantification of APOE secretion by astrocyte and neuron populations. **E** Analysis of calcium transients shows slow calcium transients in the separated astrocytes compared to neuron populations. *n* = 3~5; *p < 0.05, **p < 0.01, ***p < 0.001, ****p < 0.0001.

To dissect if the observed *APOE2* phenotypes were mediated through neurons, astrocytes, or both, we sought to generate cultures that consist exclusively of neurons or astrocytes. In this vein, we employed a magnetic activated cell sorting (MACS) protocol targeting an established astrocyte-restricted cell surface marker, CD44, to deplete neural cultures of astrocytes [40, 41] (Fig. 3A). Briefly, hiPSC-derived hNPCs were differentiated to mixed neural cultures, dissociated, and MACS-separated into CD44−positive and –negative populations. Upon separation, we analyzed both populations for levels of CD44 expression using flow cytometry. As expected, the presumptive neuron population was negative for the CD44 surface maker while the isolated astrocyte population was 92–99% CD44−positive (Fig. 3B). Importantly, the relative CD44 levels were consistent between independent differentiations and MACS isolations (Supplementary Fig. S5). Further analysis of the isolated cell populations demonstrated the CD44−negative neuronal population expressed robust levels of neuronal marker TUJ1 and mature neuron markers MAP2 and NEUN (Fig. 3C**;** Supplementary Fig. S6a). On the other hand, CD44−positive astrocytes lacked expression of neuronal markers and displayed immunoreactivity for the astrocytic maker S100β (Fig. 3C**;** Supplementary Fig. S6b).

To characterize the transcriptional profile of these isolated neuron and astrocyte populations we performed RNA-sequencing (RNA-seq) analysis (Supplementary Fig. S7). Analysis of the genes upregulated in the CD44−positive astrocytic population revealed high expression of astrocyte-specific markers (e.g., *CD44*, *VIM*, *S100A16*, *TIMP1*, *LIF*, *OSMR*, *PLD1*, *FKBP5*, *SDC4*) that regulate processes related to astrocyte function including extracellular matrix secretion and remodeling, cytokine release, and inflammatory response (Supplementary Fig. S7a) [42–47]. By comparison, expression of these markers was largely absent in the CD44−negative neuronal population, which expressed high levels of neuronal specific-makers (e.g., *MAP2, RBFOX3, SYP, DLG4, SLC17A6, GRIN1, GRIN2B, GRIN2D*) associated with neuronal function including cytoskeletal protein regulation, synapse formation, and neurotransmitter release (Supplementary Fig. S7a) [48–51]. In addition, gene ontology (GO) analysis further confirmed the identity of these cell populations (Supplementary Fig. S7b). Specifically, the CD44−negative neuronal population was enriched for genes related to neuronal biological processes (e.g., synaptic signaling, neurotransmitter exocytosis), cellular components (e.g., axon, dendrite), and molecular functions (e.g., ion channel activity, neurotransmitter binding) (Supplementary Fig. S7b, left panel). Likewise, GO-analysis confirmed that the CD44−positive astrocyte population contained gene sets related to canonical astrocytic biological processes (e.g., cytokine response, immune system processes), cellular components (e.g., focal adhesion, extracellular matrix), and molecular functions (e.g., extracellular matrix adhesion, cytokine binding) (Supplementary Fig. S7b, right panel). Importantly, correlation analysis revealed a high degree of transcriptional similarity between cells isolated from independent differentiations and MACS separations (Supplementary Fig. S7c).

Although the CD44−positive and –negative cell populations expressed the genes and proteins typical of astrocytic and neuronal populations, respectively, we next wanted to confirm the functional identity of these cell types. As it relates to APOE, astrocytes are the primary producers of APOE in the central nervous system (CNS), although it has been shown that neurons also produce APOE to a lesser degree [5, 52]. Therefore, we measured the amount of secreted APOE in the medium in the astrocytic and neuronal cultures. Indeed, we confirmed that levels of APOE levels secreted by the astrocyte cultures was significantly higher compared to purified neuronal populations (Fig. 3D). Finally, to evaluate functional differences between the neuron and astrocyte populations, we assessed spontaneous calcium transients in pure cultures. Consistent with functional characteristics of neurons and astrocytes, we found that astrocytes exhibited slow calcium transients with longer periods compared with the rapid, frequent firing of neurons, further confirming cellular identity [53] (Fig. 3E**;** Supplementary Videos 1–6). Collectively, these data demonstrate that our MACS-based protocol allows for the efficient separation of functionally mature astrocytic and neuronal sub-populations.

**Fig. 4:**
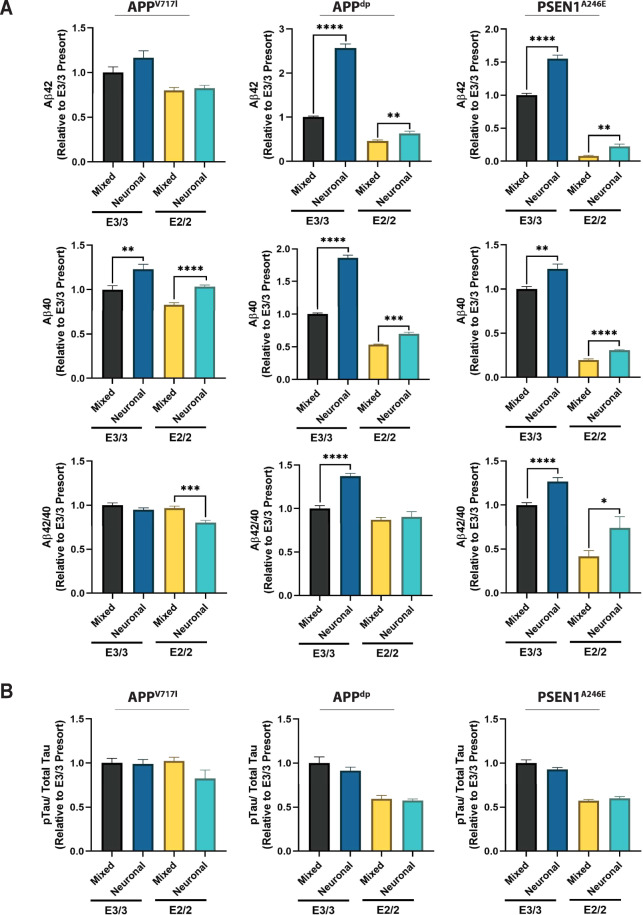
Amyloid and phosphorylated-tau (p-tau) levels in astrocyte-depleted cultures. **A** Quantification of secreted Aβ in mixed and pure neuronal cultures. **B** Quantification of the proportion of phosphorylated tau in levels in mixed and pure neuronal cultures. *n* = 6; *p < 0.05, **p < 0.01, ***p < 0.001, ****p < 0.0001.

Next, we wanted to assess the contribution of the astrocyte populations on the AD-related phenotypes observed in the isogenic pairs. Thus, we measured the levels of secreted Aβ in the mixed neuron-astrocyte cultures and cultures that consisted exclusively of neurons. Across all three isogenic pairs, we found that levels of secreted Aβ40 were significantly higher in the neuron only cultures compared to the mixed cultures, underscoring the role of astrocytes in amyloid clearance via uptake and degradation (Fig. 4A, middle panels). This result is consistent with previous studies employing mouse cell lines in the context of co-culture systems that have demonstrated the importance of astrocytes regulating neuronal *APP* expression levels [54]. Moreover, we found that removal of astrocytes led to elevated levels of secreted Aβ42 in the APP^dp^ and PSEN1^A246E^ neuron cultures but not in the APP^V717I^ cultures (Fig. 4A, upper panels). Likewise, the relative Aβ42/40 ratio was only increased in the pure neuronal cultures derived from the APP^dp^ and PSEN1^A246E^ (Fig. 4A, lower panels). Notably, the levels of phosphorylated tau were unaffected by removal of astrocytes from the mixed cultures (Fig. 4B), providing further evidence that pathological phosphorylation of tau is governed by a mechanism independent to increased amyloid. Together, these findings suggest that APOE2 modulates the secreted amyloid profile of neurons, in part, through interactions with the astrocytes.

### Differences in AD phenotypes cannot be explained by differences in astrocyte Aβ uptake or receptor expression profile

**Fig. 5:**
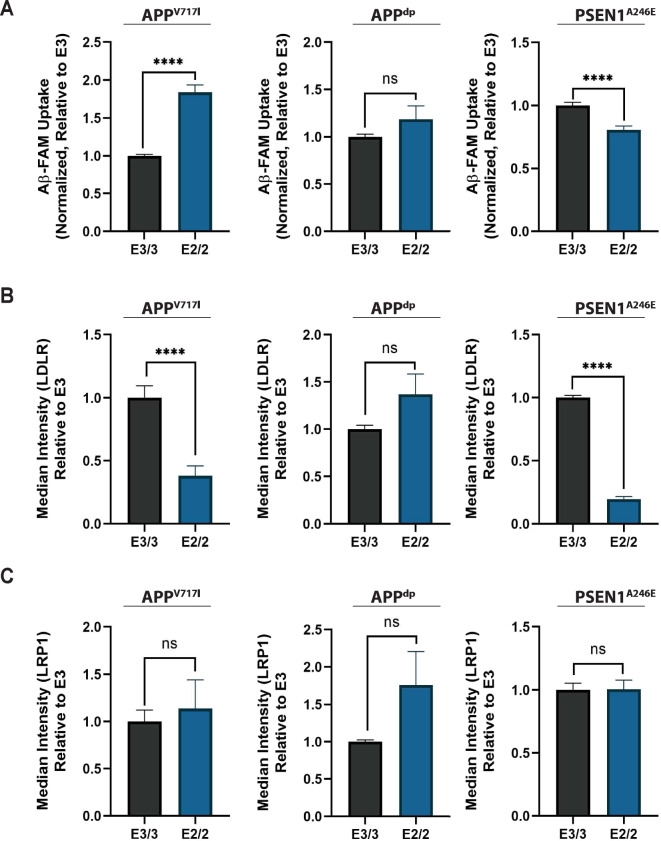
Characterization of Aβ uptake and cell-surface receptor expression in pure astrocytic populations. Quantification of internalized FAM labelled Aβ42 (*n* = 14~20 from 2–3 independent differentiations) (**A**), LDLR expression (**B**), and LRP1 expression (**C**) in pure astrocytic populations using flow cytometry (*n* = 8~11 from 2–3 independent differentiations); *p < 0.05, **p < 0.01, ***p < 0.001, ****p < 0.0001.

We next wanted to explore the possible mechanisms by which astrocytes could reduce Aβ levels in *APOE2* mixed cultures. It has been well documented that astrocytes play a critical role in regulating the extracellular levels of Aβ through multiple clearance mechanisms [55]. Moreover, numerous studies have shown that APOE influences AD pathology by regulating Aβ uptake by astrocytes [56, 57]. In fact, recently it was shown that *APOE4* hiPSC-derived astrocytes exhibit compromised Aβ uptake when compared with *APOE3* astrocytes [58]. Therefore, we speculated that *APOE2* astrocytes uptake Aβ more efficiently than *APOE3* astrocytes, which could potentially account for the reduction in Aβ levels seen in our *APOE2* isogenic mixed cultures. To test this hypothesis, we treated purified astrocytes from isogenic pairs with FAM-labelled Aβ42 for 24 h. After 24 h, cells were washed and treated with trypsin to remove surface-bound Aβ. The levels of internalized FAM-Aβ were then measured by flow cytometry. As expected, astrocytes internalized a significantly greater amount of Aβ (Supplementary Fig. S8a) compared to neurons across all cell lines. In addition, our analysis revealed that compared to neurons, astrocytes did not secrete significant levels of Aβ42 or Aβ40, consistent with previous studies with primary astrocytes [59, 60] (Supplementary Fig. S8b). However, of all the cell lines analyzed, only *APOE2* astrocytes harboring the APP^V717I^ mutation showed a significant increase in Aβ uptake compared to *APOE3* astrocytes (Fig. 5A, Supplementary Fig. S3c).

Previous work has shown that Aβ can be cleared by astrocytes through indirect or direct association with APOE in the context of several receptors mainly LDL receptor-related protein 1 (LRP1) and LDL receptor (LDLR) [61–64]. In particular, work has shown that suppression of LRP1 reduced amyloid uptake, suggesting uptake may be dependent on LRP1 [57, 65]. To assess differences in surface expression of LRP1 and LDLR between isogenic pairs, we compared fluorescence intensity of isogenic astrocytes stained with fluorescently labeled antibodies for LDLR and LRP1 (Fig. 5B-C, Supplementary Fig. S3C). This analysis revealed that in three of four lines examined there was a statistically significant decrease in LDLR expression in *APOE2* astrocytes (Fig. 5B, Supplementary Fig. S3c) and no significant difference in LRP1 expression between any of the isogenic pairs (Fig. 5C, Supplementary Fig. S3c). We next examined if there was a correlation between the levels of Aβ uptake and the expression levels of LDLR and LRP1 (Supplementary Fig. 9). When examining all cell lines, regardless of *APOE* genotype, mutation type, and disease status, we observed a slight negative correlation between Aβ uptake and LDLR expression and a modest positive correlation between Aβ uptake and LRP1 expression (Supplementary Fig. 9a). Further probing these relationships revealed that these correlations only reached levels of significance when considering mutation type or disease status (Supplementary Fig. 9b) and not *APOE* genotype (Supplementary Fig. 9c). In sum, this data indicates that the reduction of Aβ levels observed in the *APOE2* cultures compared to *APOE3* cultures cannot be attributed solely to differences in astrocytic uptake of secreted Aβ or related receptors.

### *APOE2* alters Aβ profile through changes in APP processing

**Fig. 6:**
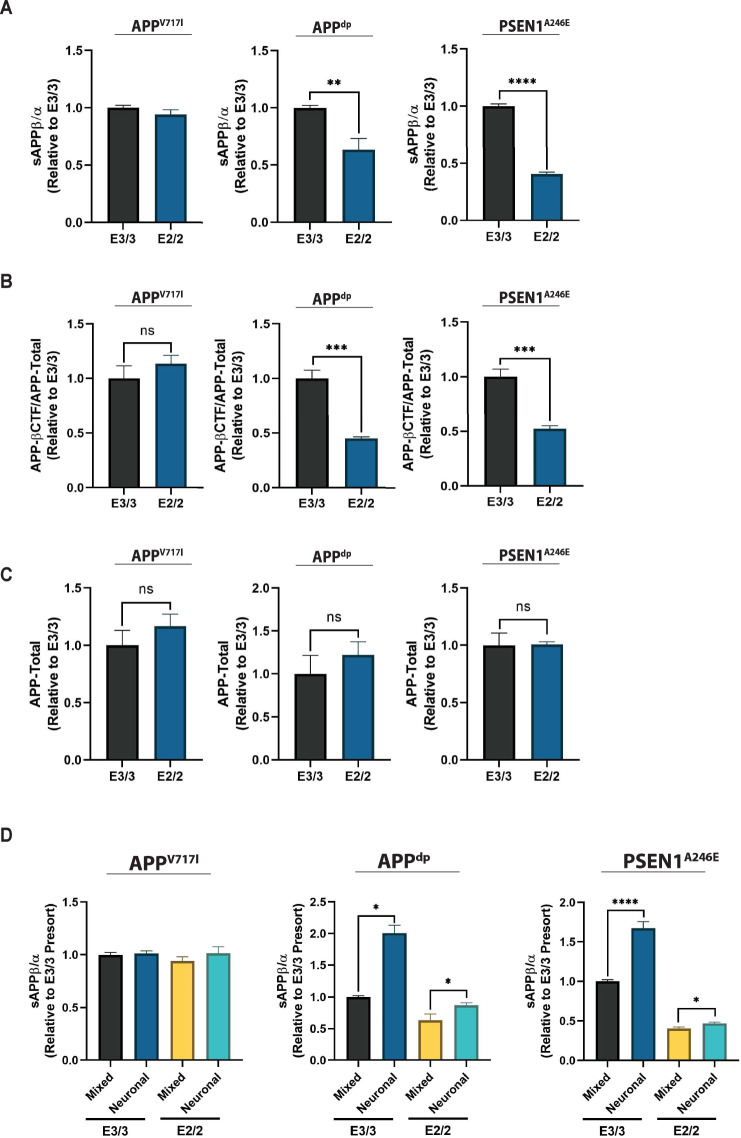
*APOE2* alters amyloidogenic processing of APP. **A** Quantification of sAPPβ and sAPPα fragments in hiPSC-derived neural cultures (*n* = 12~15 from 3 independent differentiations). **B** Quantification of the ratio of APP-βCTF to total protein in cell lysate (*n* = 4). **C** Quantification of total APP protein normalized to total protein in lysate (*n* = 4~8). **D** Comparison of sAPPβ and sAPPα levels in mixed and pure neuronal cultures (*n* = 3~15). *p < 0.05, **p < 0.01, ***p < 0.001, ****p < 0.0001.

Because we did not observe any *APOE* genotype-dependent effects on Aβ uptake we wanted to examine potential mechanisms by which *APOE2* might modulate production of Aβ. In particular, we hypothesized that the reduction of Aβ levels observed in the *APOE2* cultures could be attributed to differences in amyloidogenic versus non-amyloidogenic processing of APP. In the amyloidogenic processing of APP, β-secretase cleavage of full-length APP results in the generation of the membrane-bound C-terminal fragment (CTF-β) and extracellular release of soluble APPβ (sAPPβ). Subsequent processing of CTF-β by γ-secretase results in the generation of amino-terminal APP intracellular domain (AICD) as well as Aβ species. Conversely, α-secretase cleavage of full length APP via the non-amyloidogenic pathway results in the release of soluble APPα (sAPPα) and CTF-α, which precludes Aβ production [66]. Therefore, to investigate how *APOE2* may affect these processes in our isogenic cell culture system, we compared the levels of soluble APP (sAPP) fragments secreted into culture medium generated by APP cleavage due to the pathogenic β-secretase (sAPPβ) or non-pathogenic α-secretase (sAPPα) pathway. Comparing the ratio of sAPPβ/sAPPα in isogenic mixed cultures, we observed that the ratio was reduced in APP^dp^ and PSEN1^A246E^ *APOE2* cultures relative to *APOE3* cultures (Fig. 6A). However, the effect was more modest in APP^V717I^ cells (Fig. 6A), perhaps due to strong mutation-specific effect on amyloidogenic processing of APP [24] that cannot be entirely mitigated by *APOE2*. In agreement with these findings, we also found that the levels of CTF-β relative to total APP were also reduced in APP^dp^ and PSEN1^A246E^ *APOE2* cultures relative to *APOE3* cultures (Fig. 6B). In addition, there was no significant difference in total APP levels between isogenic pairs suggesting that the increased sAPPβ/sAPPα ratio and CTF-β levels were likely attributed to a shift from amyloidogenic to non-amyloidogenic processing and simply not reduction in total APP (Fig. 6C). To further investigate if this effect was mediated by the presence of astrocytes in cultures, we measured sAPPβ/sAPPα ratios in purified neuronal cultures. This analysis revealed an increase in the levels of sAPPβ relative to sAPPα across lines, consistent with the increase in Aβ peptides observed upon astrocyte removal in neuronal cultures (Fig. 6D). Overall, a reduction in the ratio of sAPPβ/sAPPα suggests a shift from amyloidogenic to non-amyloidogenic processing when *APOE3* is converted to *APOE2*. Given that astrocyte populations did not display significant Aβ production relative to neurons (Supplementary Fig. S8b) [67, 68], this consistent trend suggests a protective function of APOE2 involving the modulation of APP processing in neurons that might drive the isoform-specific reduction in Aβ in *APOE2* cultures.

## Discussion

It has been well-established that polymorphism in the *APOE* gene is one the strongest genetic predictors of sporadic Alzheimer’s disease (AD) risk [69]. In particular, *APOE2* is linked to a significantly reduced likelihood of AD onset and progression as well as increased life span [3, 70]. As such, it has been suggested that mimicking the protective effects of *APOE2* is a viable therapeutic intervention [71]. Nonetheless, with much of the field focused on the risk-inducing effects of *APOE4*, the mechanisms of the protective effect of *APOE2* have not been extensively studied [3, 71]. In particular, studies using isogenic hiPSC lines to study *APOE* function have all focused on elucidating toxic functions of *APOE4*, leaving mechanisms governing *APOE2* protective function largely unexplored [52, 72, 73]. In this study, we utilized three hiPSC lines derived from patients with early onset familial AD (fAD) caused by three independent autosomal mutations, including one of the most common point mutations in APP (APP^V717I^), a point mutation in PSEN1 (PSEN1^A246E^), and a duplication in APP (APP^dp^). Previous studies have demonstrated that relative to neural cultures derived from non-demented control hiPSC lines that neural cells generated from these lines display robust AD-related phenotypes including elevated levels of Aβ and phosphorylated tau [21, 22, 24]. To allow for the identification of phenotypic differences attributed solely to *APOE* genotype, we used genome editing to generate matched *APOE2* lines from each *APOE3* parental line. To our knowledge, this is the first isogenic hiPSC-derived culture system that allows for the study of the effects of *APOE2* on disease-related phenotypes.

Analysis of mixed neuronal-astrocytic cultures derived from *APOE* isogenic pairs revealed that conversion of *APOE3* to *APOE2* dramatically reduced secreted Aβ levels. These data are consistent with evidence seen clinically where *APOE2* is associated with milder AD pathology including significantly reduced amyloid deposition in the neocortex, as well as animal studies that suggest the *APOE2* allele markedly reduces brain amyloid pathology in Alzheimer’s disease mouse models [11, 74, 75]. Further, these data suggest that conversion of *APOE3* to *APOE2* was sufficient, at least in part, to reduce levels of Aβ to those observed in cells derived from non-demented control (NDC) hiPSCs. Interestingly, we did not observe a difference in Aβ levels in isogenic cultures derived from NDC hiPSCs. This suggests that the protective effects of APOE2 are only observed in the context of fAD cultures with elevated Aβ species and cannot be readily observed in context of NDC cultures that do not have significantly elevated Aβ levels. Moreover, analysis of the Aβ42/40 ratio in neural cultures revealed a reduced ratio in the *APOE2* cells harboring the PSEN1^A246E^ mutations but no difference between isogenic pairs with the APP^dp^ and APP^V717I^. Although it has been postulated that elevated ratios of Aβ42:Aβ40 directly result in the pathological amyloid plaques found in AD patients [66, 76] we speculate that differences in this ratio observed across cell lines may be a result of fAD mutation-specific differences in amyloid species generation. In particular, previous studies with the APP^V717I^ line have demonstrated that this mutation leads elevated levels of Aβ42, but not Aβ40, compared to cells derived from non-demented controls [24]. Therefore, the modulating effect of *APOE2* on Aβ42 and Aβ40 observed in this line may not be sufficient to lower the Aβ42:Aβ40 ratio. Overall, the reduction in total amyloid mediated by the *APOE2* genotype seen across all three isogenic pairs, coupled with other studies that have shown that APOE mediates Aβ aggregation in an isoform-specific manner (APOE2 < APOE3 < APOE4) [77], suggests a potential mechanism by which *APOE2*-dependent modulation of amyloid production coupled with APOE2 isoform reducing aggregation of amyloid, may work in concert to prevent severe neuron dysfunction [78].

Broadly speaking, it has been reproducibly shown that AD patients that are carriers of *APOE2* have significantly reduced cortical Aβ deposition [11], while the effect of *APOE2* on tau pathology has not been as conclusive [10, 79, 80]. In our analysis, we show that only cultures with altered Aβ42/40 ratio also showed alterations in levels of pathogenic tau in response to conversion of *APOE3* to *APOE2*. Our results linking these changes in Aβ and p-tau closely parallel new evidence from Kim and colleagues that provide strong evidence that tau pathology is tightly correlated with Aβ42/40 ratio alone, and not total Aβ levels as previously thought [39]. Further, in an independent study using hiPSC-derived neurons, it was demonstrated that abrogating amyloid production using small molecules had no effect on levels of tau phosphorylation in cultured neurons [52]. Taken together, this suggests that the modulation of tau pathology by *APOE2* might be driven solely by the effects on Aβ42/40 ratio and not increased amyloid generation.

In the central nervous system, neurons act as the primary producers of Aβ whereas non-neuronal cells, such as astrocytes, facilitate its clearance [8]. The imbalance in these processes is thought to be a primary driver of Aβ oligomerization and subsequent formation of amyloid plaques found in AD patients [66, 76]. However, the role of APOE2 in Aβ generation and extracellular removal is not well known [5]. As it relates to ApoE and Aβ uptake by astrocytes, previous studies with immortalized [57, 81] and hiPSC-derived [58] astrocytes have shown that *APOE4* astrocytes display reduced Aβ uptake when compared with *APOE3* astrocytes. Therefore, we speculated that *APOE2* astrocytes might exhibit a gain-of-protective function by enhancing Aβ uptake in our mixed cultures. However, we only observed higher levels of Aβ uptake in purified *APOE2* astrocytes with the APP^V717I^ mutation. Interestingly, we when examined the relationship between levels of Aβ uptake and LDLR/LRP1 receptor expression we observed only mutation- and not *APOE*-genotype specific correlations. This is consistent with other studies that have shown LRP1 expression can have fAD mutation-dependent expression patterns [82, 83]. Likewise, other groups have reported that although LDLR is responsible for Aβ-uptake in astrocytes, this occurs independent of ApoE [61]. Alternatively, other studies have shown compared to *APOE3* that *APOE4* astrocytes have reduced surface expression of LRP1 leading to impaired Aβ uptake [81]. Therefore, we speculate that effect of ApoE isoform on Aβ uptake in astrocytes is driven primarily by mutation-specific effects on receptor expression. Future studies in which expression of these receptors are experimentally modulated (i.e., knockdown, overexpression) in the context of *APOE* isogenic lines with various fAD-related mutations will need to be performed to further probe these relationships. With respect to neurons, recent work has shown that LDLR and LRP1 facilitate the endocytosis of tau and its subsequent spread [84, 85]. In this regard, future studies that examine the ApoE isoform-dependent relationship between neuronal expression of LDLR/LRP1 and tau uptake would be of interest in elucidating the effects of *APOE2* on the mitigation of AD-related phenotypes.

With regards to the effects of ApoE and Aβ production by neurons, ApoE has been found to elevate APP transcription and resultant Aβ generation in cultured neurons in the order E4 > E3 > E2 [23]. However, the isoform-specific effects of ApoE on amyloidogenic processing of APP have not been extensively studied. To that end, we investigated if altered amyloidogenic processing could be implicated in the reduction of Aβ species observed in the *APOE2* mixed cultures. Soluble APP fragments are an indirect measurement of pathogenic processing and sAPPβ and CTF-β peptides have been shown to increase in fAD neurons [86–88]. Our analysis of secreted amyloidogenic sAPPβ relative to non-amyloidogenic sAPPα as well as CTF-β revealed a significant reduction in pathogenic processing in APP^dp^ and PSEN1^A246E^ *APOE2* cultures compared to *APOE3* cultures. While this trend was also seen in APP^V717I^ cells, it was much less pronounced compared to the other two cells lines. It was been documented that the APP^V717I^ mutation is located within the transmembrane region of APP which preferentially increases amyloidogenic β-secretase cleavage at levels that exceed other fAD-related mutations [24]. Therefore, we contend that the significantly elevated mutation-specific levels of sAPPβ reduced the protective *APOE2* effect size in APP^V717I^ cultures relative to the other two cell lines. In addition, we observed that although the removal of the astrocyte subpopulation led to increased levels of pathogenic sAPPβ, congruent with our observation of increased Aβ, this did not diminish the effect of *APOE2* on APP processing. This suggests that both cell autonomous and cell non-autonomous aspects contribute the effect of *APOE2* on APP processing by neurons. Currently, the specific mechanisms by which *APOE2* alters APP processing are unclear. A variety of mechanisms regulate amyloidogenic versus non-amyloidogenic processing of APP, including but not limited to the following: (i) cytoplasmic phosphorylation of APP, (ii) levels of activity of kinases implicated in APP phosphorylation, (iii) expression and activity levels of enzymes (i.e., α-, β, and γ-secretase) involved in APP cleavage, and (iv) intracellular APP trafficking [89–91]. In particular, ApoE has been shown to form a physical association with APP, causally altering Aβ output by potentially acting as a molecular chaperone for APP processing [57, 92]. In addition, blocking this interaction could abrogate the isoform specific effects of APOE4 on Aβ levels [92, 93]. Therefore, we speculate that APOE2 might directly affect how APP is presented to APP cleavage enzymes, resulting in alterations in APP processing and Aβ secretion profiles. However, future studies will be required to resolve the particular mechanisms that enable this potential gain-of-protective effect of *APOE2*.

In conclusion, our findings using isogenic hiPSC-derived neurons provide some of the first characterization of *APOE2*-dependent changes in a hiPSC cell culture model. In particular, we choose to examine these *APOE2* effects in the context of fAD lines that have consistently displayed robust AD-related phenotypes [21, 22, 24]. Remarkably, introduction of *APOE2* into hiPSCs with fAD-related mutations significantly mitigated the strong disease-related phenotypes typically observed in the neural cultures derived from these cell lines. Even though disease-relevant phenotypes in neural cells generated from sporadic AD (sAD) hiPSCs have been highly variable or largely absent [5], future studies that employ *APOE* isogenic sAD hiPSCs will be necessary to increase the translation of these findings to LOAD. Finally, although we did not interrogate all the potential hypothesized mechanisms by which *APOE2* modulates AD-risk, we uncovered clues that will set the stage for more detailed studies. In particular, *APOE2* had a strong mitigating effect on Aβ production likely through a mechanism related to modulation of amyloidogenic processing of APP. Collectively, these results open up potential new targets to mimic the protective effects of *APOE2* to alleviate AD onset and disease-related progression.

## Supplementary information

Supplemental Video 1

Supplemental Video 2

Supplemental Video 3

Supplemental Video 4

Supplemental Video 5

Supplemental Video 6

Supplemental Material
