# Supplementary material for: APOE2 mitigates disease-related phenotypes in an isogenic hiPSC-based model of Alzheimer’s disease
Source: Mol Psychiatry. 2021 Apr 9;26(10):5715–32. doi: 10.1038/s41380-021-01076-3 (PMC8501163; doi:10.1038/s41380-021-01076-3)
Supplement: Supplementary file 7 — Supplemental Material [file 41380_2021_1076_MOESM7_ESM.pdf]

SUPPLEMENTAL FIGURE LEGENDS

Supplemental Figure 1. Characterization of isogenic *APOE2* fAD hiPSC line with APP^{dp} mutation. (**a**) Immunofluorescence analysis of pluripotency markers OCT4, SOX2, NANOG. (**b**) Flow cytometry staining for pluripotency marker TRA1-81. An isotype control (Alexa Fluor 647 Mouse IgM, κ) was used for generating gates. (**c**) Karyotype analysis of edited hiPSCs. (**d**) Trilineage differentiation of *APOE2* hiPSCs and immunofluorescence staining of endoderm (AFP), mesoderm (SMA), and ectoderm (TUJ1) markers. (**e**) Sanger sequencing of the *APOE* locus confirming a cysteine at amino acid position 158.

Supplemental Figure 2. Comparison of neuronal marker expression across differentiations. Representative immunofluorescence staining of mixed neuronal cultures from independent differentiations for neuronal marker TUJ1 and mature neuronal markers MAP2 and NEUN.

Supplemental Figure 3. Amyloid and phosphorylated-tau (p-tau) levels in non-demented control (NDC) cultures. (a) Quantification of secreted soluble Aβ levels and **(b)** Tau protein levels in NDC neuronal mixed cultures. (n=4) ; * = p<0.05

Supplemental Figure 4. Comparison of Aβ release between neural cultures derived from Alzheimer's disease (AD) and non-demented control (NDC) hiPSCs. Quantification of secreted soluble Aβ levels in AD neural cultures relative to those in NDC E3/3. n=4~15 from 1-3 independent differentiations; * = p<0.05, ** = p<0.01, *** = p<0.001, **** = p<0.0001.

Supplemental Figure 5. CD44 expression profile of purified fAD neurons and astrocytes across differentiations. Flow cytometry plot overlay of purified neurons and astrocytes from two independent differentiations stained for CD44 surface protein, gated for corresponding isotype controls.

Supplemental Figure 6. Additional characterization of purified neuronal and astrocytic populations. (**a**) Representative immunofluorescence staining of purified neurons for neuronal marker TUJ1. (**b**) Representative immunofluorescence staining of purified astrocytes for astrocytic maker S100β.

Supplemental Figure 7. Transcriptional profile of fAD isogenic neurons and astrocytes. (a) Comparison of the genes differentially expressed by neurons and astrocytes normalized to the maximum FPKM within each isoform, including several cell type specific markers in focus. **(b)** Gene ontology terms for biological process, cell component and molecular function upregulated in pure-neuron and -astrocyte cultures. **(c)** Heatmap depicting the Pearson correlation coefficients for gene expression levels between fAD neurons and astrocytes.

Supplemental Figure 8. Aβ uptake and secretion levels in purified neuronal and astrocytic populations. (a) Flow cytometry analysis of labeled FAM-Aβ42 peptide internalization in isogenic neurons and astrocyte (n=3 for neurons, n=14~20 for astrocytes). **(b)** Quantification of secreted Aβ in astrocytes compared to paired isogenic neurons (n=3 for astrocytes, n=11~15 for neurons). * = p<0.05, ** = p<0.01, *** = p<0.001, **** = p<0.0001.

Supplemental Figure 9. Relationship between Aβ uptake and LDL receptor expression in isogenic fAD and NDC astrocytes. (a) Flow cytometry based median intensity measurement of dextran normalized Aβ uptake (y axis) is plotted against surface expression of LDLR/LRP1 (x axis) receptors with a linear regression line (x-y pairs n=78). **(b)** Linear regression line of Aβ

uptake (y axis) and LDLR/LRP1 expression (x axis – top/bottom) for each cell line (x-y pairs n= 22 [APP^{V717I}] , 22 [APP^{dp}], 16 [$PSEN1^{A246E}$], 18 [NDC]). **(c)** APOE isoform-specific linear regression line of Aβ uptake (y axis) and LDLR/LRP1 expression (x axis – top/bottom) for fAD astrocytes (x-y pairs n=30). Spearman correlation coefficient (r) and p values are reported on all plots.

SUPPLEMENTAL VIDEO LEGENDS

Supplemental Video 1. Calcium imaging for astrocytes and neurons derived from *APOE3* APP^{V717I} hiPSCs.

Supplemental Video 2. Calcium imaging for astrocytes and neurons derived from *APOE2* APP^{V717I} hiPSCs.

Supplemental Video 3. Calcium imaging for astrocytes and neurons derived from *APOE3* APP^{dp} hiPSCs.

Supplemental Video 4. Calcium imaging for astrocytes and neurons derived from *APOE2* APP^{dp} hiPSCs.

Supplemental Video 5. Calcium imaging for astrocytes and neurons derived from *APOE3* $PSEN1^{A246E}$ hiPSCs.

Supplemental Video 6. Calcium imaging for astrocytes and neurons derived from *APOE2* $PSEN1^{A246E}$ hiPSCs.

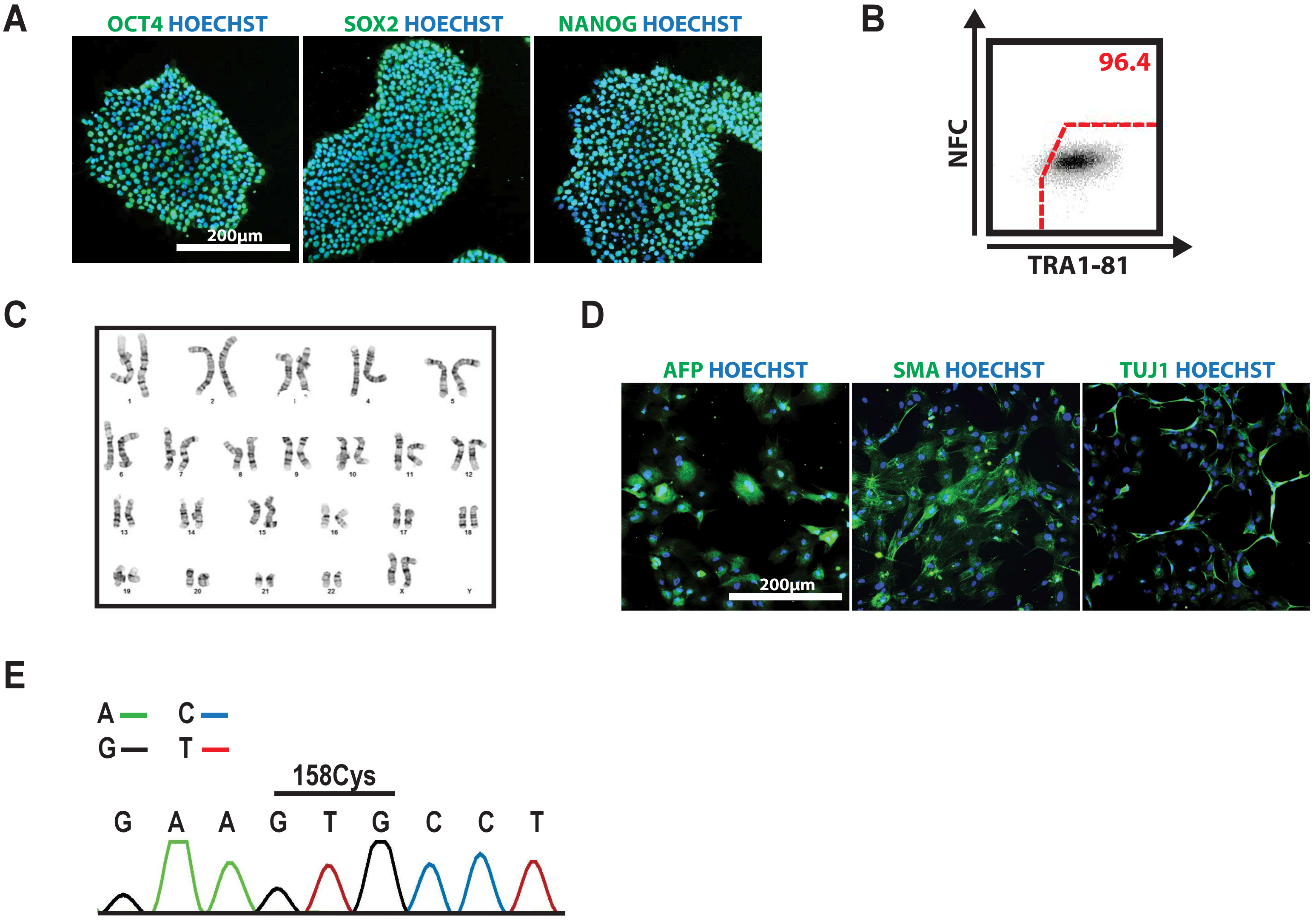
A
OCT4 HOECHST
SOX2 HOECHST
NANOG HOECHST
200µm
B
96.4
NFC
TRA1-81
C
D
AFP HOECHST
SMA HOECHST
TUJ1 HOECHST
200µm
E
A
C
G
T
158Cys
G A A G T G C C T

Figure S1

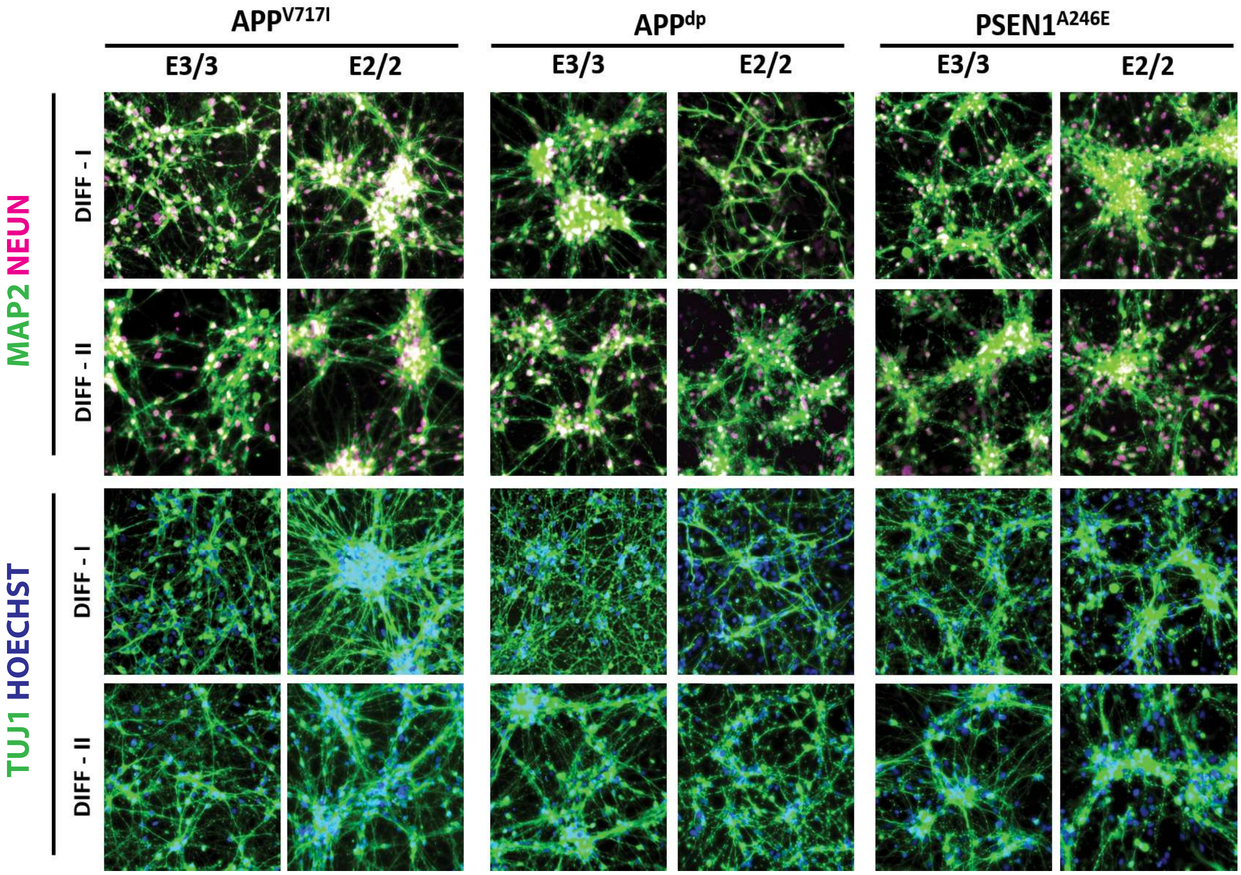
APP^{V717I}
APP^{dp}
PSEN1^{A246E}
E3/3
E2/2
E3/3
E2/2
E3/3
E2/2
MAP2 NEUN
DIFF - I
DIFF - II
TUJ1 HOECHST
DIFF - I
DIFF - II

Figure S2

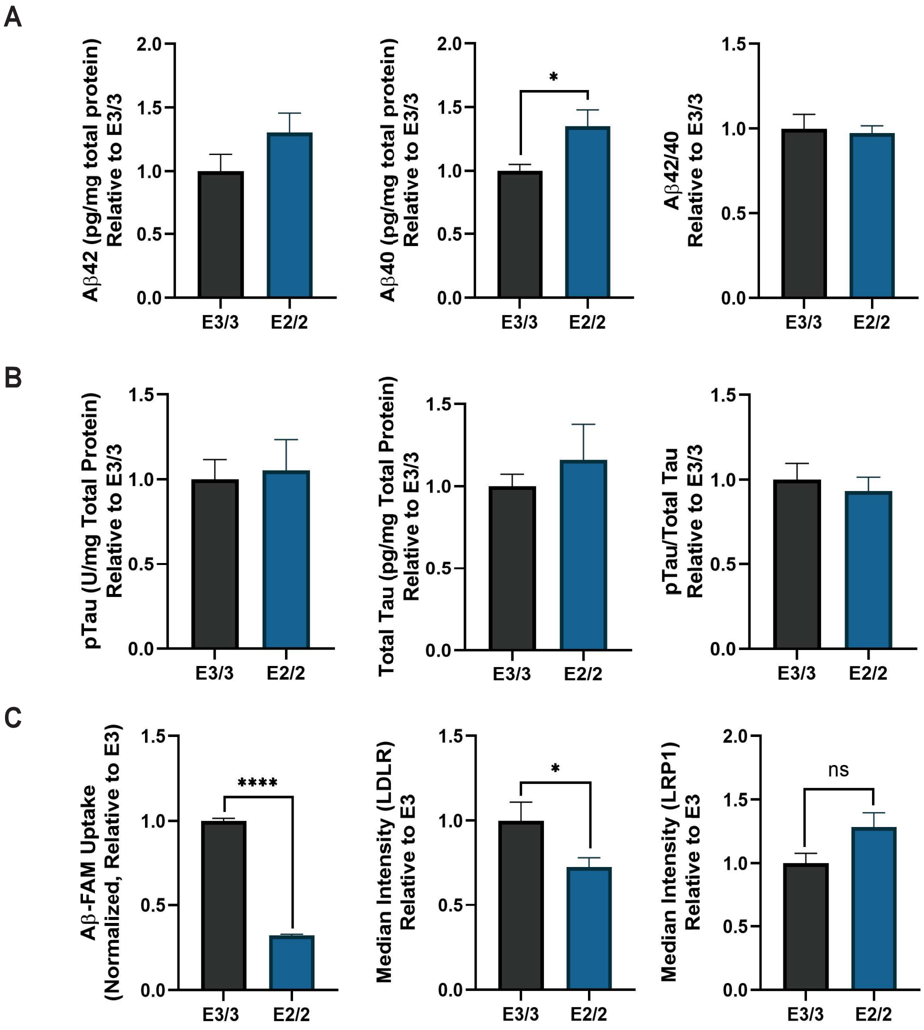
A
Aβ42 (pg/mg total protein) Relative to E3/3
2.0
1.5
1.0
0.5
0.0
E3/3
E2/2
Aβ40 (pg/mg total protein) Relative to E3/3
*
Aβ42/40 Relative to E3/3
B
pTau (U/mg Total Protein) Relative to E3/3
Total Tau (pg/mg Total Protein) Relative to E3/3
pTau/Total Tau Relative to E3/3
C
Aβ-FAM Uptake (Normalized, Relative to E3)
****
Median Intensity (LDLR) Relative to E3
*
Median Intensity (LRP1) Relative to E3
ns

Figure S3

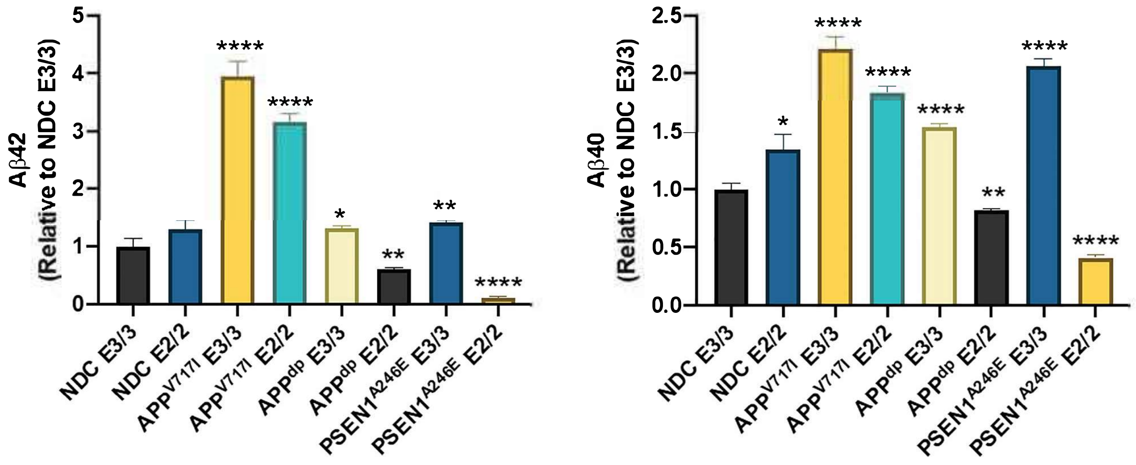

Aβ42
(Relative to NDC E3/3)
****
****
*
**
**
****
NDC E3/3
NDC E2/2
APPV717I E3/3
APPV717I E2/2
APPdp E3/3
APPdp E2/2
PSEN1A246E E3/3
PSEN1A246E E2/2
Aβ40
(Relative to NDC E3/3)
*
****
****
****
**
****
****

Figure S4

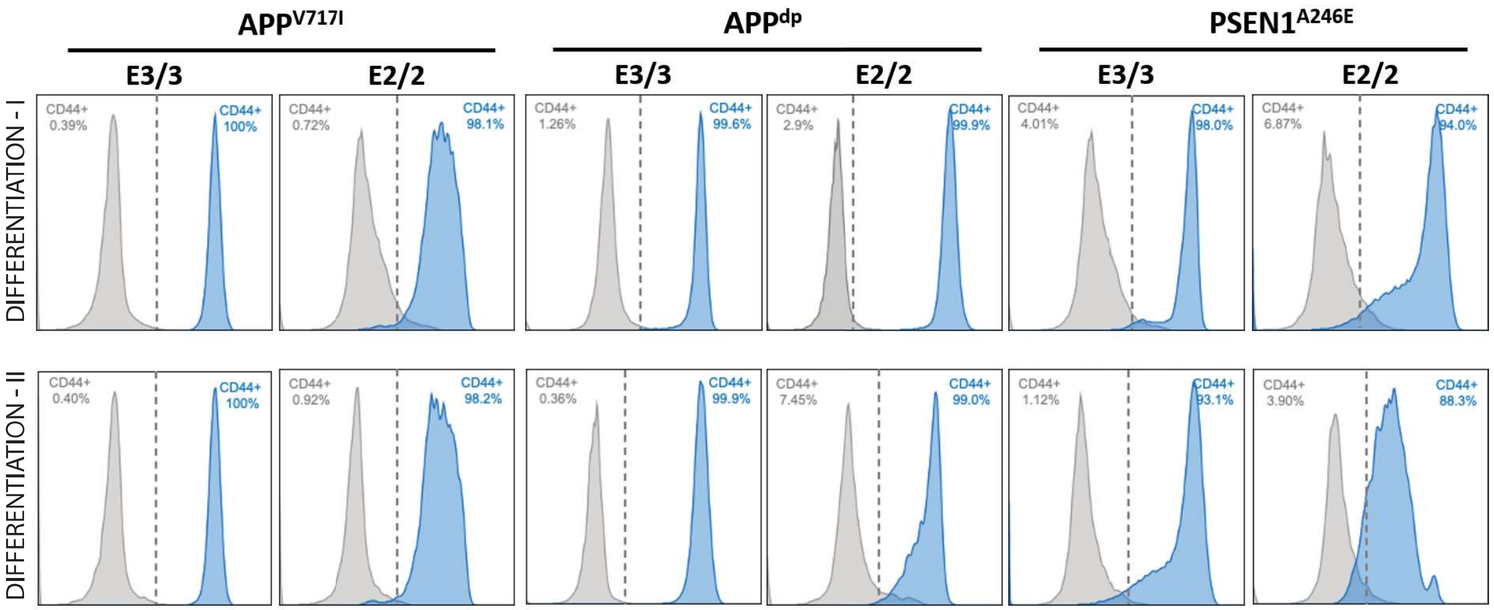
APPV717I
APPdp
PSEN1A246E
E3/3
E2/2
E3/3
E2/2
E3/3
E2/2
DIFFERENTIATION - I
DIFFERENTIATION - II
CD44+ 0.39%
CD44+ 100%
CD44+ 0.72%
CD44+ 98.1%
CD44+ 1.26%
CD44+ 99.6%
CD44+ 2.9%
CD44+ 99.9%
CD44+ 4.01%
CD44+ 98.0%
CD44+ 6.87%
CD44+ 94.0%
CD44+ 0.40%
CD44+ 100%
CD44+ 0.92%
CD44+ 98.2%
CD44+ 0.36%
CD44+ 99.9%
CD44+ 7.45%
CD44+ 99.0%
CD44+ 1.12%
CD44+ 93.1%
CD44+ 3.90%
CD44+ 88.3%

Figure S5

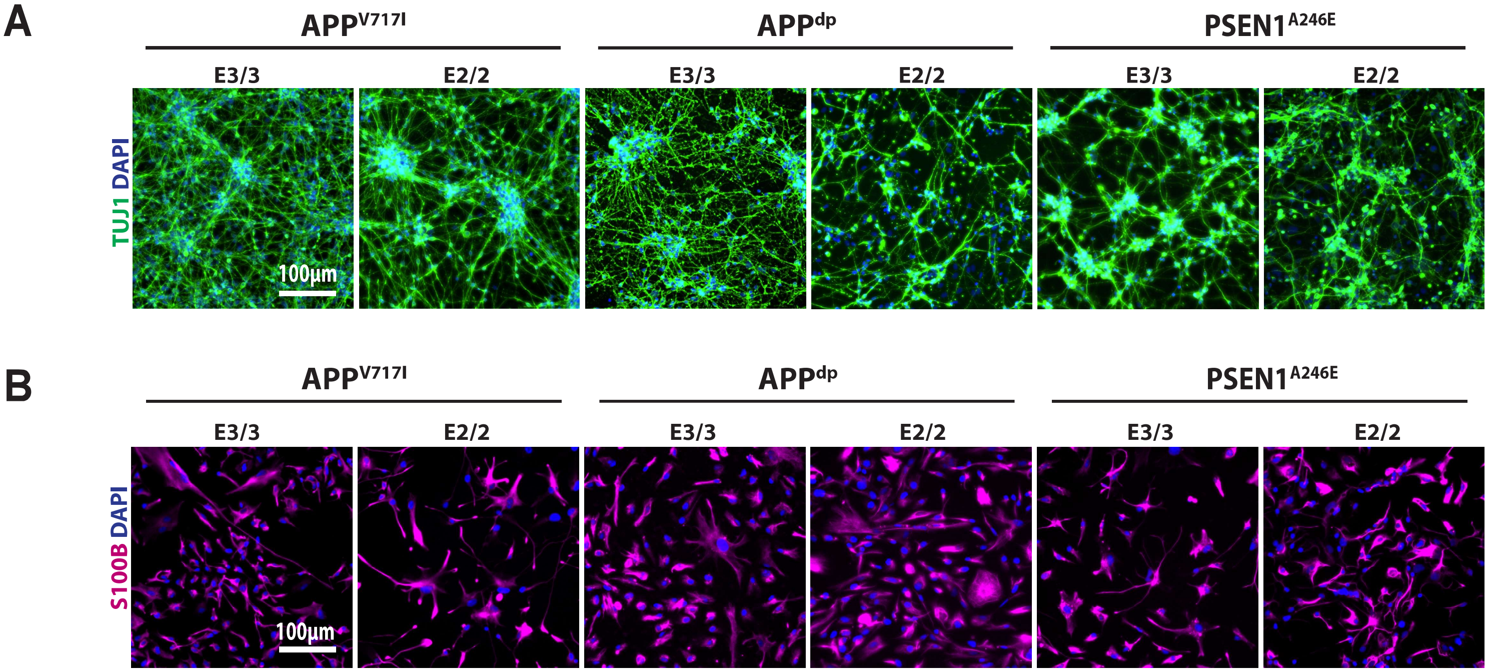
A
APPV717I
APPdp
PSEN1A246E
E3/3
E2/2
E3/3
E2/2
E3/3
E2/2
TUJ1 DAPI
100µm
B
APPV717I
APPdp
PSEN1A246E
E3/3
E2/2
E3/3
E2/2
E3/3
E2/2
S100B DAPI
100µm

Figure S6

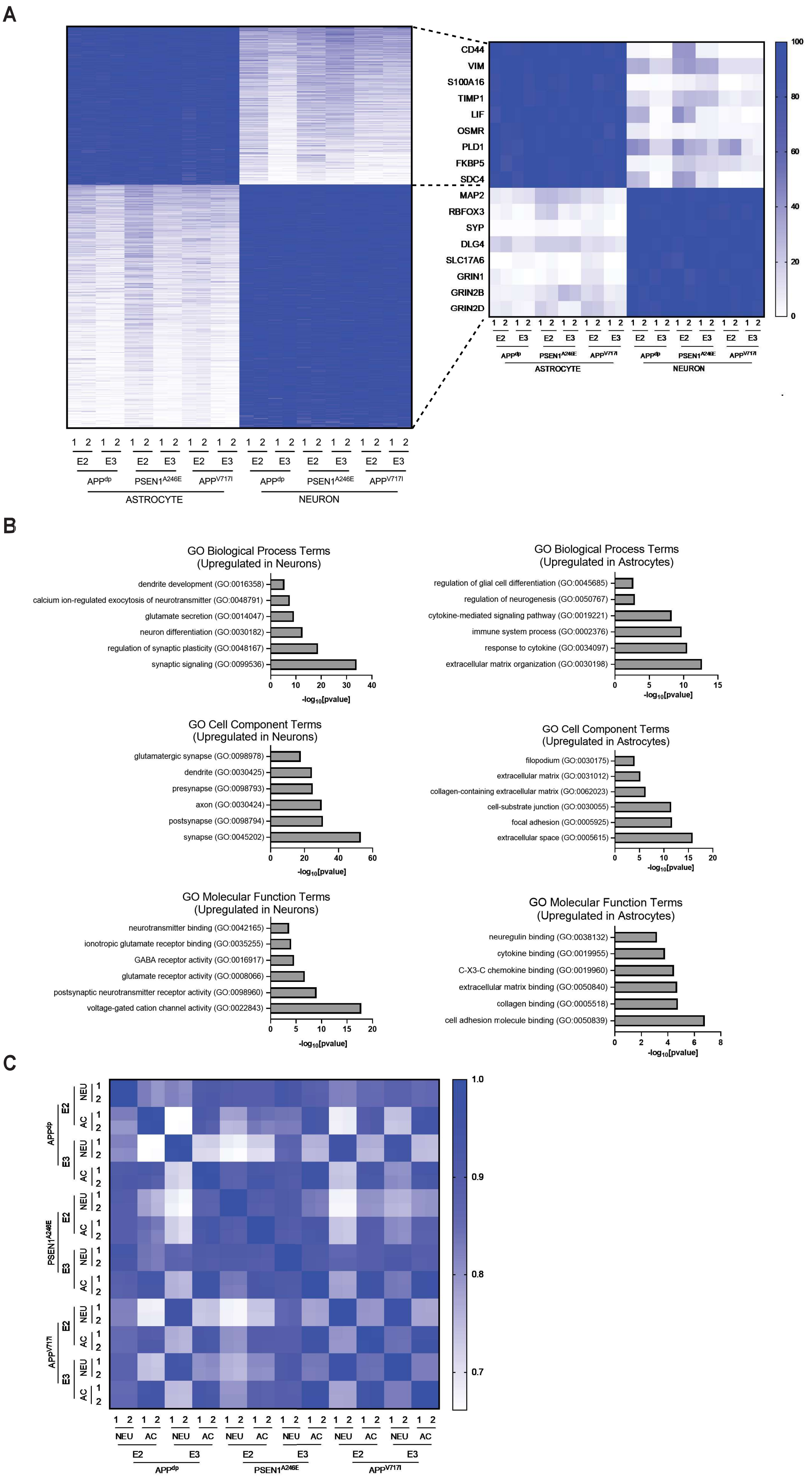
A
CD44
VIM
S100A16
TIMP1
LIF
OSMR
PLD1
FKBP5
SDC4
MAP2
RBFOX3
SYP
DLG4
SLC17A6
GRIN1
GRIN2B
GRIN2D
100
80
60
40
20
0
E2
E3
APP^{dp}
$PSEN1^{A246E}$
APP^{V717I}
ASTROCYTE
NEURON
B
GO Biological Process Terms (Upregulated in Neurons)
dendrite development (GO:0016358)
calcium ion-regulated exocytosis of neurotransmitter (GO:0048791)
glutamate secretion (GO:0014047)
neuron differentiation (GO:0030182)
regulation of synaptic plasticity (GO:0048167)
synaptic signaling (GO:0099536)
$-log_{10}$[pvalue]
GO Biological Process Terms (Upregulated in Astrocytes)
regulation of glial cell differentiation (GO:0045685)
regulation of neurogenesis (GO:0050767)
cytokine-mediated signaling pathway (GO:0019221)
immune system process (GO:0002376)
response to cytokine (GO:0034097)
extracellular matrix organization (GO:0030198)
GO Cell Component Terms (Upregulated in Neurons)
glutamatergic synapse (GO:0098978)
dendrite (GO:0030425)
presynapse (GO:0098793)
axon (GO:0030424)
postsynapse (GO:0098794)
synapse (GO:0045202)
GO Cell Component Terms (Upregulated in Astrocytes)
filopodium (GO:0030175)
extracellular matrix (GO:0031012)
collagen-containing extracellular matrix (GO:0062023)
cell-substrate junction (GO:0030055)
focal adhesion (GO:0005925)
extracellular space (GO:0005615)
GO Molecular Function Terms (Upregulated in Neurons)
neurotransmitter binding (GO:0042165)
ionotropic glutamate receptor binding (GO:0035255)
GABA receptor activity (GO:0016917)
glutamate receptor activity (GO:0008066)
postsynaptic neurotransmitter receptor activity (GO:0098960)
voltage-gated cation channel activity (GO:0022843)
GO Molecular Function Terms (Upregulated in Astrocytes)
neuregulin binding (GO:0038132)
cytokine binding (GO:0019955)
C-X3-C chemokine binding (GO:0019960)
extracellular matrix binding (GO:0050840)
collagen binding (GO:0005518)
cell adhesion molecule binding (GO:0050839)
C
1.0
0.9
0.8
0.7
NEU
AC

Figure S7

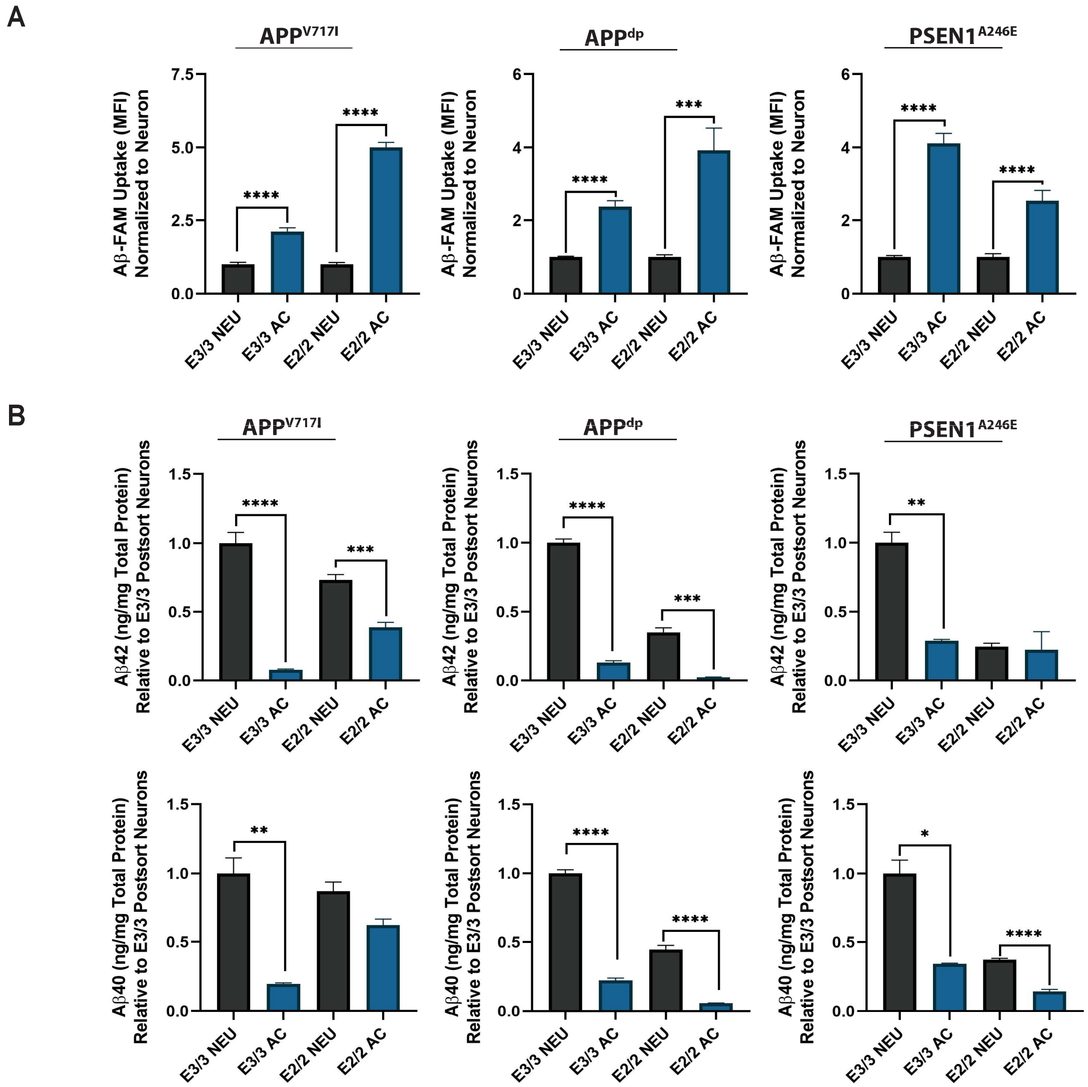
A
APP^V717I
APP^dp
PSEN1^A246E
Aβ-FAM Uptake (MFI) Normalized to Neuron
E3/3 NEU
E3/3 AC
E2/2 NEU
E2/2 AC
B
Aβ42 (ng/mg Total Protein) Relative to E3/3 Postsort Neurons
Aβ40 (ng/mg Total Protein) Relative to E3/3 Postsort Neurons

Figure S8

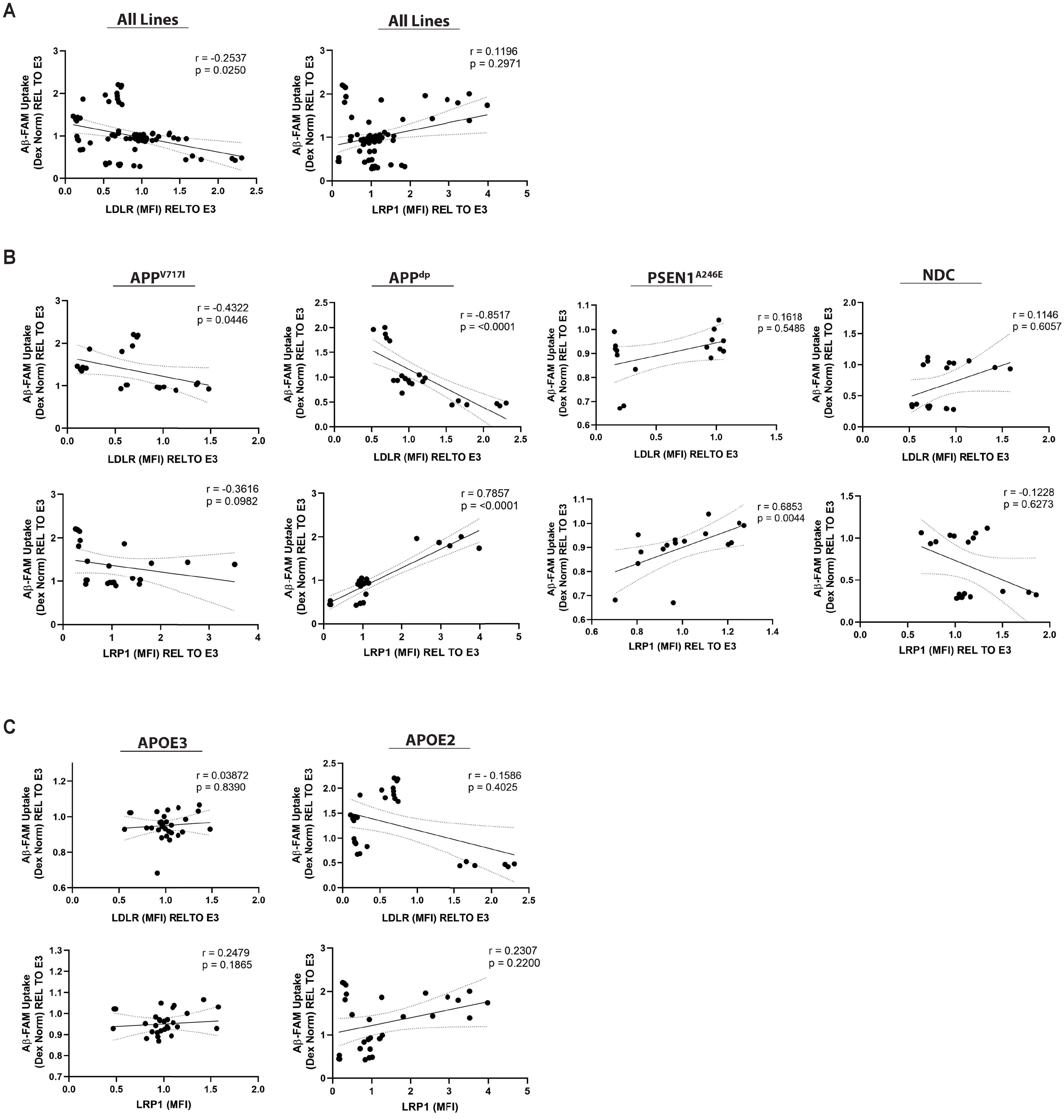
A
All Lines
r = -0.2537
p = 0.0250
LDLR (MFI) RELTO E3
Aβ-FAM Uptake (Dex Norm) REL TO E3
All Lines
r = 0.1196
p = 0.2971
LRP1 (MFI) REL TO E3
B
APPV717I
r = -0.4322
p = 0.0446
r = -0.3616
p = 0.0982
APPdp
r = -0.8517
p = <0.0001
r = 0.7857
p = <0.0001
PSEN1A246E
r = 0.1618
p = 0.5486
r = 0.6853
p = 0.0044
NDC
r = 0.1146
p = 0.6057
r = -0.1228
p = 0.6273
C
APOE3
r = 0.03872
p = 0.8390
r = 0.2479
p = 0.1865
LRP1 (MFI)
APOE2
r = - 0.1586
p = 0.4025
r = 0.2307
p = 0.2200

Figure S9
